# The Role of Mindfulness in Reducing the Adverse Effects of Childhood Stress and Trauma

**DOI:** 10.3390/children4030016

**Published:** 2017-02-28

**Authors:** Robin Ortiz, Erica M. Sibinga

**Affiliations:** 1 Departments of Internal Medicine and Pediatrics Johns Hopkins Hospital, 1800 Orleans Street, Baltimore, MD 21231, USA, Robin.Ortiz@jhmi.edu; 2 Johns Hopkins School of Medicine, 733 North Broadway, Baltimore, MD 21205, USA

**Keywords:** trauma, mindfulness, adverse childhood events, resilience, MBSR, mind-body, ACEs, at-risk youth, childhood adversity, toxic stress, allostatic load

## Abstract

Research suggests that many children are exposed to adverse experiences in childhood. Such adverse childhood exposures may result in stress and trauma, which are associated with increased morbidity and mortality into adulthood. In general populations and trauma-exposed adults, mindfulness interventions have demonstrated reduced depression and anxiety, reduced trauma-related symptoms, enhanced coping and mood, and improved quality of life. Studies in children and youth also demonstrate that mindfulness interventions improve mental, behavioral, and physical outcomes. Taken together, this research suggests that high-quality, structured mindfulness instruction may mitigate the negative effects of stress and trauma related to adverse childhood exposures, improving short- and long-term outcomes, and potentially reducing poor health outcomes in adulthood. Future work is needed to optimize implementation of youth-based mindfulness programs and to study long-term outcomes into adulthood.

## 1. Introduction

The evidence continues to mount that exposure to adverse experiences during childhood has the potential to increase morbidity and mortality both during childhood and across the lifespan into adulthood [1]. Adverse childhood experiences (ACEs) are stressful for children, and include neglect; physical, sexual or emotional abuse; exposure to violence, mental illness, incarceration, or substance abuse in the family; parental absence due to divorce or separation; and low socioeconomic status. Further, significant, traumatic, recurrent, and/or prolonged stress may have a cumulative toxic effect on the child [2,3]. In addition to the psychological toll, stress and toxic stress effect the body through increased allostatic load, the physiologic burden of such stress that may manifest as neuroanatomical changes, increased levels of inflammation, and dysfunction of the hypothalamic-pituitary-adrenal axis [4]. More recent findings are emerging suggesting that resilience, i.e., successful management of and coping with stress, can mitigate the negative consequences of such trauma [5,6]. Findings such as these have sparked a call to action for pediatricians to both recognize that, “many adult diseases should be viewed as developmental disorders that begin early in life and that persistent health disparities associated with poverty, discrimination, or maltreatment could be reduced by the alleviation of toxic stress in childhood” and “to serve as both front-line guardians of healthy child development and strategically positioned, community leaders to inform new science-based strategies that build strong foundations for educational achievement, economic productivity, responsible citizenship, and lifelong health” [2]. This is an emphatic call to work together to enhance the prevention of ACEs, provide early and accessible interventions, and broadly expand the delivery of trauma-informed care. Mindfulness is an evidence-based intervention that supports these important responses to ACEs, fundamentally enhancing self-regulation and resilience in everyday life and in the face of stress and trauma. 

This review aims to identify the benefits of mindfulness-based interventions as an approach to mitigating the negative sequelae of childhood trauma by summarizing relevant research in adult and pediatric populations. Additionally, the adaptations for introducing and teaching mindfulness for children and youth will be reviewed. Finally, future directions in the research and clinical realms related to trauma-informed mindfulness interventions will be suggested. 

## 2. ACEs and Trauma

**Figure 1:**
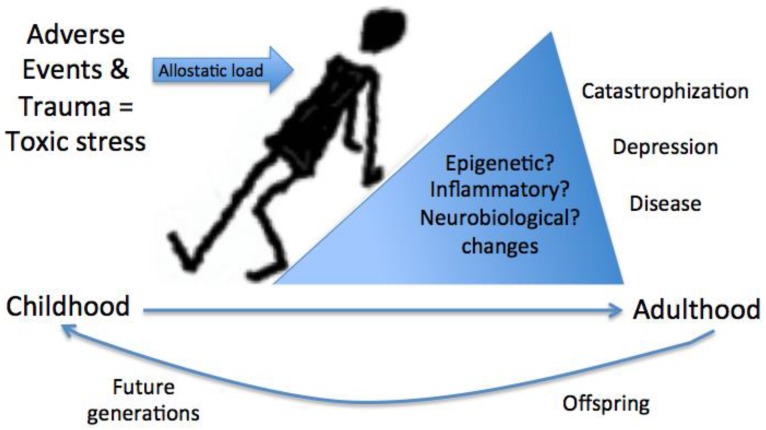
The impact of stress and trauma in childhood. Adverse childhood events, stress, and trauma contribute to toxic stress. Toxic stress that results from prolonged exposure to stress, aggregated trauma experiences, or incidents of significant emotional impact yields an increased allostatic load on the body. Allostatic load, measured by biological markers of disease risk including inflammatory cytokines, neurobiological changes, metabolic abnormalities, and epigenetic modifications, may carry over into future generations.

Research suggests that children are often exposed to significant environmental stressors and situational adversities [7]. A Centers for Disease Control and Prevention (CDC) report from five states in 2009 showed that 69% of respondents (*n* = 26,229) report at least one adverse childhood event (ACE) with 9% experiencing up to five adversities [7]. A smaller study of children found 34% of those screened in school report exposure to at least one trauma and evidence of post-traumatic stress even without a current diagnosis [8]. One of the first studies of ACEs (including traumas such as abuse, neglect, witnessing violence against mother, substance use in the home, household mental illness, parental separation or divorce, or household member incarcerated) showed that not only do ACEs cluster together, but there is a dose response relationship with overall health, such that larger numbers of ACEs are associated with poorer health [1]. Other adversities associated with a risk for exposure to trauma include low socioeconomic status and the associated lower education [9] and risky family environments [10,11]. Such negative experiences in the absence of a protective buffer may manifest as toxic stress, yielding an increased allostatic load to the body causing prolonged activation of physiologic stress responses, which over time may yield poor health outcomes and future illness [3,4,12]. For example, ACEs have been associated with biological markers of disease risk including inflammatory cytokines, metabolic abnormalities, and epigenetic modifications [4,13,14] (Figure 1). Remarkably, such epigenetic modifications may carry across generations, as identified in the glucocorticoid receptor related gene *FKBP5* of holocaust survivors also found in their offspring [15]. Toxic stress can result from different situations, including single stressors that are prolonged in exposure (such as recurrent emotional abuse), multiple stressors that become toxic when aggregated (such as low socioeconomic status), living below the poverty line and having limited educational opportunity, and/or traumatic experiences of greater emotional intensity or severity (such as sexual abuse).

Specific ACEs, such as those associated with an adverse living environment limited in support and opportunity, have been shown to be associated with negative health outcomes. Such an adverse environment encompasses socioeconomic factors (such as household income, education of parents, and occupational prestige of parents), and risky family environment (such as living with a household member with a substance use disorder, mental illness or history of incarceration, living in a chaotic or disorganized environment, or experiencing violence and lack of parental warmth) [10]. Low socioeconomic status (SES) is associated with reduced access to educational support, or parental involvement in education [16]. Similarly, household chaos is associated with sensitive and harsh parenting, both of which predict childhood misconduct [17]. Low SES also seems to be associated with developmental delay (specifically, a delay in cognitive development of executive functioning including working memory, inhibitory control, and cognitive flexibility), poor conduct, and callous behaviors [17,18,19]. These lead to consequences of transition to adulthood such that lower SES and early life stress influence both cognitive and associated neurobiological development, which are also associated with poor health outcomes in adulthood and comorbid metabolic and cardiovascular dysfunction [20,21]. Further, adverse living environment, may predispose to the development of characteristics and behaviors that are risk factors for multiple comorbidities, including obesity, smoking, and increased blood pressure trajectory, throughout adolescence into adulthood, and are further specifically associated with metabolic dysfunction and cardiovascular disease in adulthood [10,22,23,24]. Risky family environments have also been associated with dysfunctional emotional processing, mood disorders, and hypothalamic-pituitary-adrenal (HPA) axis dysfunction [25].

There is diversity in both the types of traumatic experiences that may exist in childhood and the broad systems that may be affected. ACEs are associated with increased poor and risky health behaviors including substance use, smoking, risky sexual activity, and sedentary lifestyle in adulthood. ACEs also show a graded relationship with the presence of mental health disorders, adult ischemic heart disease, cancer, chronic lung disease, skeletal fractures, and liver disease in a study of 9508 Americans in 1998 [1]. In addition, ACEs have also been associated with alcoholism [26], chronic obstructive pulmonary disease [27], autoimmune disease [28], quality of life [29], drug use [23], risk for intimate partner violence [30], sexually transmitted diseases [31], suicide attempts [32], maladjustment and misconduct in and outside of school, as well as risk for incarceration [33,34]. Importantly, many of these conditions begin early in childhood and are reversible or preventable by mitigating risk factors. 

Studies have also identified associations between childhood adversity and trauma and specific adult diagnoses including fibromyalgia [35], migraine [36], irritable bowel syndrome [37], insomnia and insufficient sleep [38], cancer [39], cognitive function in mental health disorders [40], as well as learning and behavior problems and obesity in youth [41] and adolescent pregnancy and fetal death [42]. Collectively, this research suggests the significant and pervasive negative impact of childhood ACEs and trauma on short- and long-term health outcomes, and therefore, the necessity and opportunity to prevent and intervene on ACEs, and to provide trauma-informed services broadly to offset the negative consequences in children, the adults they will become, and possibly even future generations. 

## 3. Mindfulness

Mindfulness has origins as a Buddhist concept increased through meditation that has been cultivated into a Western practice of present-focused, non-judgmental awareness [43]. Mindfulness instruction has also been offered through structured training programs such as that developed by Jon Kabat-Zinn in 1979 to enhance non-judgmental attention to the experience of the present moment, entitled Mindfulness Based Stress Reduction (MBSR) [43]. The MBSR program has been shown to increase self-reported mindfulness among participants [44,45]. However, all individuals have the capacity for mindfulness, i.e., non-reactivity, awareness, focus, attention, and nonjudgment [45], though there is variability in the amount and quality of mindfulness among individuals. Therefore, both structured MBSR and unstructured mindfulness practices outside of the MBSR model, may enhance the beneficial characteristics associated with mindfulness. 

Structured mindfulness training is available through Kabat-Zinn’s MBSR, which typically consists of eight weekly two-and-a-half-hour classes and a full-day retreat, in which participants learn a variety of formal and informal mindfulness practices, learn and discuss the mind-body connection, and have group discussions regarding the challenges to integrating mindfulness into one’s life. For particular populations, other programs have been adapted from MBSR, such as Mindfulness-Based Cognitive Therapy (MBCT), shown to be effective in reducing depression recurrences, and one focused on addiction triggers, Mindfulness Based Relapse Prevention (MBRP) [46,47]. Unstandardized mindfulness may also be offered in other formats such as aspects of educational sessions, art therapy, group therapy, yoga, or other mind-body interventions. Though this review will focus on standardized MBSR and MBSR-related programs to highlight evidence for their practice, there will be brief discussion of the practice and implications of other mindfulness delivery methods.

### 3.1. Mindfulness in Trauma

**Figure 2:**
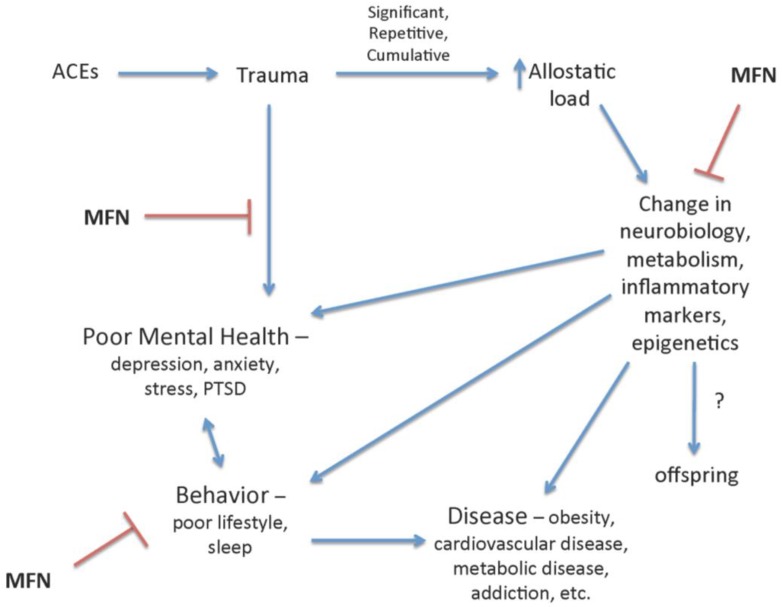
The negative impact of adverse childhood events (ACEs) and trauma in childhood is reduced by mindfulness (MFN). Mindfulness has been shown to mitigate the psychological, behavioral, and physiological changes associated with ACEs and trauma and increased allostatic load. MFN, specifically, reduces symptoms of depression and posttraumatic stress disorder (PTSD) associated with stress and trauma, and is inversely associated with poor health behavior and biological makers of metabolic, neurologic, and inflammatory dysfunction and disease. Stress has been demonstrated to be associated with epigenetic modifications that may persist in offspring; therefore, mindfulness interventions may reduce these negative influences.

Mindfulness instruction has been shown to benefit individuals with a known trauma or ACE. Mindfulness may do this by both an indirect effect of negating the acute response to trauma and stress, but also by inhibiting underlying consequences of chronic exposure to stress and trauma such as psychiatric, metabolic and cardiovascular disease through the influence on lifestyle choices, underlying biochemistry and neurobiology (Figure 2). In 50 women exposed to trauma including witnessing family violence, experiencing childhood physical or sexual abuse, or sudden loss of a loved one, an 8-week MBSR program was associated with decreased symptoms of stress and trauma exposure including perceived stress, depression, trait and state anxiety, emotion dysregulation, and posttraumatic stress symptoms [48]. Additionally, 27 female survivors of sexual abuse in childhood, experienced significantly reduced symptoms of depression, posttraumatic stress disorder (PTSD), and anxiety after an 8-week MBSR intervention [49]. This beneficial effect continued at follow-up 2.5 years later [50], which highlights that mindfulness may have an effect on the formation of related psychiatric comorbidities. This was demonstrated in two populations at risk for high rates of trauma and/or ACEs and related psychiatric disease. In a group of incarcerated women (*n* = 33) improvements in perceived stress, anxiety and depression were seen with a 12-week mindfulness intervention [51]. In a small qualitative study of a population of survivors (*n* = 12) of political violence, when mindfulness was combined with art therapy in a unique 4-day intensive program, themes of resilience emerged [52]. 

Research also suggests a role for mindfulness to mitigate consequences of toxic stress, by identifying benefit in individuals exposed to high stress environments by both enhancing long-term coping, and influencing the related physiologic effects of stress on the HPA axis. Klatt et al. studied intensive care unit workers finding that a mindfulness intervention was linked to increased resilience [53]. In active duty military personnel preparing for deployment, mindfulness was shown to mitigate the response to stressful experience [54], which suggests that it may both reduce current stress experience and predispose to enhanced coping prior to stress exposure. However, mindfulness, additionally, has lasting effects beyond exposure to prolonged stress as demonstrated by numerous studies in veteran populations and those exposed to war and bereavement. Veterans (*n* = 58) who participated in an 8-week long mindfulness intervention including a day long retreat, saw clinically significant improvements in their Post Traumatic Stress Disorder (PTSD) symptoms up to 17 weeks afterward, and another study suggests a shorter duration (*n* = 62, 4-session program) intervention may also decrease PTSD symptoms though only studied up to 8-weeks post [55,56]. The same group found that mindfulness also decreased cortisol levels in the veterans [57]. To suggest that this may be a global effect, other studies have supported these similar findings. A study that used mindfulness through mantra based meditation also showed decreased PTSD symptoms in veterans [58]. In one of the largest mindfulness studies of the veteran population, a mediation program that elicited mindfulness as an outcome, also reduced PTSD symptoms in 391 veterans in a uniquely implemented Department of Veteran Affairs meditation program through six medical centers [59]. In veterans with mental health comorbidities mindfulness improved sleep quality [60].

These underlying influences of mindfulness may mitigate mental health outcomes, enhance quality of life, and reduce somatic symptoms. A short 4-session intervention incorporating mindfulness in the mind-body approach, modestly increased resilience and also decreased depressive and anxiety symptoms and perceived stress in veterans though only assessed post-intervention [61]. These effects translate to improvements in quality of life [62] with a reduction in somatic symptoms including dizziness, fatigue and tension in addition to depressive symptoms as shown in veterans with PTSD [63]. Undergraduates ages 18–36, who participated in a mindfulness activity interrelated with expressive writing exercises about a past stress or trauma demonstrated decreased physical symptoms, poor sleep, and negative affect; with beneficial findings apparently linked to the mindfulness component, given expressive writing alone was not predictive of improvement [64]. Mindfulness is also inversely associated with functional disability in Iraq and Afghanistan war veterans (*n* = 115) [65], and with PTSD in a trauma exposed Iranian population (*n* = 1708) [66]. It may also offer protection in burn-out and compassion fatigue, such as in a study of traumatic bereavement workers [67]. 

Further, research has shown that these beneficial effects of mindfulness may be generalizable to healthy populations, regardless of known exposure to stress and trauma. In large meta-analyses, mindfulness was shown to reduce stress in healthy adult individuals [68] and to reduce anxiety, depression, and pain in diverse clinical populations [69]. Mindfulness is inversely related to anxiety, experiential avoidance, distress and uncertainty, external stimulus reactivity (poorer executive control), and persistence of negative affect [70,71]. Mindfulness programs can improve coping and resiliency in specific conditions including quality of life in patients with multiple sclerosis [72], depression and anxiety in patients with cancer [73], and specifically in trauma-exposed individuals with human immunodeficiency virus (HIV), can reduce PTSD symptoms [74]. 

Importantly, the beneficial effects of mindfulness on psychological and somatic symptoms and foundations for disease extend beyond negating the psychological impact of stress and may influence physiologic dysfunction (Figure 2). In general, mindfulness has been modestly associated with alterations in markers of inflammation, cell-mediated immunity, and biological aging [75]. Specifically, in women with interpersonal trauma (*n* = 50), not only were their psychological symptoms improved, as discussed above, but their inflammatory cytokine, interleukin-6 (IL-6) levels decreased [48]. Mindfulness has neurobiological modification potential as demonstrated by enhanced resting state functional connectivity as well as executive control via the dorsolateral prefrontal cortex, both coinciding with decreased levels of peripheral IL-6 levels [76]. Such biomarkers may be modulated by epigenetic changes through, histone-modifying enzymes also found to be associated with mindfulness interventions [77]. These neurobiological changes may represent modifications that can influence behavior and disease risk factors in adults and possibly the predisposition to disease in children, especially those at higher risk when exposed to stress, trauma, and/or toxic stress. 

Though children may be exposed to different types of trauma then adults, these studies collectively suggest that mindfulness may serve to buffer the effects of stress and trauma in children and into adulthood. 

### 3.2. Mindfulness in Youth

Recent mindfulness studies in youth, specifically in populations with known trauma exposure or populations at high risk for ACEs suggest promise in improving a variety of outcomes, including mental health symptoms, behavior and quality of life, and coping (Table 1). In one randomized control trial (RCT) of a population of urban youth in a low-income environment, middle-school age male children (*n* = 41) underwent a 12-week school-based mindfulness intervention that resulted in decreased negative coping [78]. The group additionally yielded decreases in anxiety and rumination and possibly school related stress response as suggested by a flatter cortisol curve over the course of the study (three months) and school term compared to the attention, social experience and time matched control, health education group. A larger RCT in two urban public middle schools (*n* = 300) showed that the 12-week, school-based MBSR program led to reduced depression, self-hostility, negative affect, negative coping, rumination, somatization, and post-trauma stress symptoms [79]. Additionally, one study of mindfulness in youth in foster care, ages 14–21 (*n* = 42), showed a trend toward post-intervention changes in state anxiety, qualitative gains in-group effectiveness in social gains, and coping with stress was observed [80].

These studies with foster care youth and in urban low-resourced areas likely represent children affected by or at risk for on-going stresses, and/or trauma [78,79,80,81,82], especially illustrated by the reduction in post-stress trauma symptoms in the MBSR arm. Other studies also support the promising hypothesis that mindfulness may offer coping skills, emotional processing and resilience. Many of these studies are done in the school environment. In a RCT of 522 children ages 12–16, the mindfulness arm post-intervention outcomes included lower stress, greater well-being, and fewer depressive symptoms [83]. It is important to note that the control group in this study was a usual school curriculum and did not control for peer experience, attention, or time. In a study of 101 sixth grade children, a 6-week mindfulness curriculum decreased suicidal ideation and self-harm [84] compared to a matched experiential activity control group. Though this study did not show an effect on internalizing or externalizing problems, others have demonstrated it to improve classroom behavior, and attention [85,86] (Table 1). Mindfulness may also offset the psychiatric comorbidities accompanied by traumatic experiences that impact the lives of youth in and outside of the classroom. Biegel and colleagues showed that 104 adolescents ages 14–18 benefited with improved state anxiety, sleep, stress, self-esteem, psychiatric symptoms (such as somatization and hostility) and global assessment functioning [87].

Though mindfulness has been demonstrated to be beneficial across a variety of domains when taught directly to youth, it may also influence youth outcomes when those surround them learn mindfulness, such as teachers and parents [88]. A study by Singh and colleagues demonstrated that an intervention administered to preschool teachers resulted in decreases in difficult behaviors, decreases in negative interactions, and an increase in compliance among students [89]. Similarly, Jennings and colleagues observed that mindfulness training improved teacher well-being and characteristics of impactful teaching including improved efficacy, and reduced stress (*n* = 35) [90].

Resilience in children may also arise through mindful parenting. Less parenting stress, increased parental warmth, and increased parental attention to their children may also contribute to buffering against the poor health outcomes associated with ACEs [10,91]. Given the number of studies that suggest mindfulness may reduce parental dismissal of their children, mindful parenting may also contribute to resilience against trauma [92]. Mindfulness in parents was associated with each individual’s positive perspective on quality of the relationship in black families of stepparents and their child [93]. Mindfulness in parents also may be protective against poor psychobehavioral outcomes in children as has been demonstrated by an inverse association between internalizing and externalizing problems in youth across multiple age groups (young childhood, middle childhood, and adolescence, *n* = 215) [94]. Mindful parenting interventions may improve parent-child relationship qualities [94], but there is mixed evidence regarding the effectiveness of mindful parenting in general depending on the outcomes of interest [92]. However, one study specifically showed benefit that may be extrapolated to relate to parenting children exposed to ACEs. Parents in inner-city methadone programs demonstrated reduced ratings on the Child Abuse Potential Inventory indicating lower potential for physical abuse [95], and therefore show promise to reducing the sequelae of ACEs. 

In summary, high-quality structured mindfulness programs for youth show promise by reducing mood and emotion dysregulation (decrease depressive, self-hostility, PTSD, anxiety and negative affect symptoms), negative-coping with stress, and improved school adaptation (classroom behavior and discipline, social and academic competence), and attention, mitigating negative effects and potential exacerbations of ACEs. Mindfulness has also been shown to benefit those important to youth, including parents and teachers. 

### 3.3. Resilience and Mindfulness 

In order to understand how the practice of mindfulness might be offered as an intervention mitigating the relationship between childhood trauma and health outcomes, one must appreciate the contribution of mindfulness to enhancing resiliency [6]. Resilience is buffering, through successful coping, against adverse outcomes after exposure to traumatic experiences. Conceptually, the lack of resilience may predispose to conditions like PTSD and further unfavorable health consequences as seen in those who experience trauma. In an adult urban inner-city population resilience was found to be inversely associated with symptoms of PTSD [5] and depression, but childhood trauma was positively associated with the presence of PTSD and depression symptoms [96]. Further, there may be neurobiological underpinnings to the buffering effect of resilience on ACEs. Though ACEs have been associated with neurocognitive impairment, trait resilience is associated with better neurocognitive skills such as non-verbal memory [20,21,97]. Importantly, a limitation to exploring this concept is the assessment of resilience, which in research investigations is done by collecting data on related concepts such as perseverance and self-confidence as assessed by the Connor-Davidson-Resilience Scale (CD-RISC) questionnaire, or other concepts measures like coping, social support, emotional and behavioral reactivity, and compassion [98,99]. Nonetheless, enhancing these important domains through mindfulness may serve collectively or independently as buffers against trauma.

Mindfulness may increase resilience in those who have experienced trauma, because it offers an alternative to the common psychological dissociation that occurs after trauma, which can prevent healthy processing or coping [100]. The mindfulness characteristics of accepting without judgment, and acting with awareness are inversely associated with PTSD symptoms [101]. This is likely due to the mindfulness teaching of non-judgmental acceptance of painful and unpleasant thoughts, as well as feelings and practice of decreased reactivity to them [102]. In a cross-sectional study of 125 individuals with substance dependence and a history of trauma, the characteristic of mindfulness was inversely associated with thought suppression, which may represent a form of dissociation and which was strongly associated with PTSD symptoms [103]. Accordingly, trait mindfulness was also associated with decreased cravings. 

## 4. Mindfulness Program Considerations

### 4.1. Mindfulness Practice and Instructor Training

Mindfulness instruction as a practice has taken on many forms, but MBSR is structured as a 26-h (eight 2.5 h sessions with 1 full day) involving mindful exercises, such as body-scans, mindful breathing, and yoga with meditation instruction, group discussions and encouragement of home practice [43,104]. However, some programs have found effect with as few as 12-h of instruction, with no clear association between hours of instruction and effect size of post-MBSR effects, but studies are varied and limited in their duration of effect [105]. Britton et al. implemented a 15-min daily 6-week course in children, and Kalmanowitz et al. demonstrated that even a 4-day long intensive program in adults demonstrated benefit [52,84]. 

There is no centralized credentialing required for teaching mindfulness. While there are certificate programs to become an MBSR instructor [106], many programs have branched off from Jon Kabat-Zinn’s original model and even studies of mindfulness interventions vary in the qualifications of those implementing instruction to subjects. It is a further challenge to find practitioners that have completed both MBSR and trauma-informed training, and still further, rarely so with specific qualifications to work with youth. Importantly, even trauma therapists who offer mindfulness in practice, have varying degrees of self-practice that may impact their level of involvement [107]. 

There have been attempts at unique modifications to programming for children or those with special focused needs (Table 2 and Table 3). Some studies have specifically involved instructors who have had years of experience specifically with children [78,79,80,81,82]. One study partnered a mental health therapist with adolescents in the community to co-facilitate a mindfulness meditation practice, though the study does not comment on the level of experience of the mental health therapist with mindfulness [108]. For mindfulness training implementation in schools, many programs have branched from the classic MBSR program as described by Meiklejohn et al., 2012 [109]. For example, The Mindfulness in Schools Project (UK based), based on MBSR, is one that has been studied with successful outcomes including fewer depressive symptoms in adolescents, and the Mindful Schools (US based) improved teacher-based perceptions of classroom behavior in a 6th grade public elementary school children [110,111]. Many similar programs to The Mindfulness in Schools Project and Mindful Schools, as seen in Table 3, share in their methodology that mindfulness trained individuals lead practices in the school environment and also educate teachers to lead students [109]. 

Though many studies have administered mindfulness interventions to teachers, showing benefits for teaching work-environment and temperament [88], none have assessed associated student outcomes directly as a result of the teachers training in isolation. However, the training of teachers is a promising approach to improved classroom outcomes. One study cited by Gouda et al., 2016, showed that schoolteachers (*n* = 82) had self-reported improvements in mood as well as measures of improved emotional regulation (decreased reactivity, increased compassion) as compared to controls, though the subjects were all female [112], and another showing decreased distress [113]. A smaller study (*n* = 15) with similar outcome measures also suggested reduction in burnout [114]. Based on such studies, programs have launched to improve classroom learning environments such as the “Cultivating Awareness and Resilience in Education” Program [90]. Since, a larger combined RCT recently showed improvements in schoolteacher sleep and work satisfaction and decreased bad mood, but this was of a workplace mindfulness training not specific to educational environments [115]. Though only one small case study in preschool students showed decrease in negative social interactions [89], taken with the teacher trials, these studies suggest that it may be worth considering that future studies assess the effects and consider if training teachers alone may impact resiliency against ACEs. Therefore, perhaps implementation of programming for both students and teachers is ideal, as found by Gouda et al., 2016 in that both students and teachers showed improvement in interpersonal problems, and affect, whereas students also showed improved school-specific self-efficacy by specific developed self-assessment questionnaires [88]. It is important to keep in mind that lack of benefit of mindfulness in schools programs can be due to lack of teacher experience [116]. 

Even outside of the school, teacher-led environment, children are never cared for in isolation. It may be beneficial to consider methodology of mindfulness implementation that addresses their caretakers. Given that mindful parenting yields reduced parental stress, with increased parental warmth toward their children [10,91], suggests that mindful parenting training should also be included in interventions for youth. However, many caretakers are involved in influencing children. As examples, one intervention partnered therapists with peer instructors, and another allowed time for individualized intervention for youth (average age 11.5 years) and their parents, then partnered them for family intervention [88,108,117]. The intervention to strengthen families (*n* = 9) involved seven 2-h sessions with the first hour individualized between youth and their parents and the second hour for family training. It also allowed time for mindful parenting training with teaching and practice exercises on being mindful (attentive, reducing emotional reactivity, being less judgmental) [117], demonstrating how a holistic approach including caretakers, parents and children may be implemented. 

The type and location of intervention taught varies tremendously in the literature. As discussed and seen in Table 2, many interventions are integrated directly into school curriculum [88], but also have been offered as extracurricular, in the clinic setting (primary care and specialty), during incarceration, and to parents [81,82,85,87,95,108,119]. Some offer mindfulness only as an addition to a structured and detailed training program with other included skillsets including cognitive therapy, behavioral therapy, resilience training for healthy behaviors, and meditation [6,108,119,120]. There is no study to date that has compared settings, but variation may be dependent on population and outcome of interest. For example, if targeting improvements in mood dysfunction, this has been seen across settings by various different outcome measures for PTSD, depression and anxiety. Attention and behavior outcomes are more commonly measures in studies of school-based interventions, though importantly, there is no evidence that interventions outside of school would not produce effects in the school environment. Given the broad exposure to traumas and ACEs, and the generalizable benefits of mindfulness, mindfulness programs offered through school and/or primary care sites may be most suited to reaching the broadest population of children. 

The methods of mindfulness implementation may vary given the diversity of existing academic curricula and environments for youth. Generally, schools offer the most appropriate environment to reach a broad spectrum of children across developmental stages, diagnoses, and socioeconomic circumstances, as well as stressor exposure [116]. Incorporation of mindfulness practice directly into the school curriculum addresses the suggestion of the school environment as a means to reach broad categories of children [78,79,81,86,88]. Examples of broad reaching implementations were postulated by Britton et al. who allotted half a semester on a course to mindfulness with the second half preserved for other curriculum and a mid-year switch of groups, or by Johnstone et al., who proposes to complete a study with a 2-semester health class incorporating eight weeks of mindfulness into the class [84,121]. Kuyken et al. replaced studies in either religious studies or personal, social and health education, offering the idea that perhaps mindfulness could be offered as an elective course in the curriculum [83]. 

Though the school environment offers a promising delivery format for teaching mindfulness, other methods have been suggested. Home practice is one such model and may offer shorter study formats requiring as little as 5 min [122]. Some formats have even bridged home study with one-on-one in home training [95]. Clinic groups if advertised as a ‘social’ group rather than a ‘stress management’ group may pose another potential format [80]. To enhance participation regardless of the format studies have implemented facilitating transportation to class, reminder phone calls, and providing snacks, for example [123]. Other organizations are implementing mindfulness into women and children’s shelters and community centers [124]. 

In school, home and clinic environments, a generalized training in mindfulness allows for broad population applicability, but individualized programs also may be helpful in certain specified populations. An example of individualization of mindfulness intervention methodology for a unique trauma exposed population was accomplished by a program that identified reactivity as a problem in incarcerated individuals and tailored the program “S.T.I.C”. S.T.I.C. involves the following steps: stop, take a breath, imagine, and choose, allowing the mindfulness and attention to be directed at consequences and redirection of choices [119]. Though S.T.I.C. was individualized by targeting a specific population, personalization can also be accomplished in school or clinic settings by narrowing the target population such as by diagnosis or age. For example, one study clustered female students with any eating disorder history into a mindfulness program finding improved psychosocial outcomes relative to control by six months post-intervention [120]. Grouping by diagnosis has also been beneficial in the diagnoses of attention-deficit/hyperactivity disorder (ADHD), HIV, or psychiatric conditions [82,85,87], with incorporation of mindfulness into existing therapeutic interventions like cognitive or rehabilitation therapy, MBCT or MBRP, specifically. The RAP Club was offered exclusively to 7th and 8th grade students and catered to adolescent specific life challenges in the program design [108], whereas others incorporated simplistic terminology that can be applied to younger and older students such as, “FOFBOC: Feet on the floor and bum on chair”, and “Beditation” referring to a body scan done while lying down [116]. Finally, mindfulness technique has been applied in combination with other therapies including sound and music, touch, walking, daily routines, and even “mindful texting” to allow for the inclusion of mindfulness in regular activities with the idea of “checking in” with the self as much as possible throughout the day [125]. For translating concepts of mindfulness to children, metaphors can be helpful and many children’s books are available including those authored by Thich Nhat Hahn, a Buddhist monk who has helped bring the practice of mindfulness to western society with John Kabat-Zinn [126]. 

### 4.2. Trauma-Informed Care

Per the Substance Abuse and Mental Health Services Administration (SAMHSA) a program that is trauma-informed realizes trauma prevalence and its common adverse effects, recognizes signs and symptoms of trauma, responds with standard operating procedures, and seeks to resist re-traumatization. SAMHSA provides various examples of well-known trauma-informed intervention programs and they all include general principals of safety, connection and trustworthiness, collaboration, empowerment, and current and historical societal and cultural competency [127]. Formal mindfulness instructor training incorporates a number of these elements and lays the foundation for mindfulness as a trauma-informed care (TIC) practice. 

In trauma-informed care, an intervention may be primary (preventive), secondary (reduction of severity and acuity of consequences), or tertiary (treatment of long-term sequelae) [128]. Though it is important for clinicians involved in intervention administration to be cognizant of the above principals of TIC, this does not necessarily mean that the specific stressors or traumas need to be identified for mindfulness to have a beneficial effect, though this has not specifically studied. There is a high prevalence of stress and trauma, with up to 34% of children in school reporting exposure to at least one trauma and evidence of post-traumatic stress even without a current diagnosis [8]. Further, it is known that abuse and trauma is underreported [129] and poorly recalled [130]. Even when reported, child memory of trauma can include intrusions or inaccurate recall [131], and symptoms of PTSD are often poorly recognized by parents [132]. In studies assessing previous trauma, methods of quantification vary widely from general indication of trauma domain to specifics of traumatic event(s). Further, PTSD may mask itself through generalized somatic complaints in children including trouble sleeping, low energy, stomach pain, dizziness, and headaches [133]. Taken together, these studies suggest that one cannot rely on always and accurately identifying the presence of traumatic experience in children. Yet, mindfulness may have an impact in children whether they report a trauma exposure, or not. Trait mindfulness is associated with resilience in face of adverse events, and is associated with strong cognitive abilities, and mindfulness interventions can reduce somatic manifestations of psychiatric undertones [6,70,71,87,134,135]. In populations with high likelihood of ACEs, such as low-income urban areas, school-based mindfulness instruction provided to all students (primary prevention) has been shown to improve psychological symptoms and coping and to reduce post-trauma stress symptoms [79]. Therefore, mindfulness interventions that lead to enhanced coping and resilience can be beneficial when offered to all children regardless of specific identification of trauma exposure.

## 5. Conclusions and Future Directions

In conclusion, research has demonstrated that high-quality, structured mindfulness interventions improve mental, behavioral, and physical outcomes in youth. Further, these results in combination with the well-studied interventions in adults suggest promise in preventing the poor health outcomes associated with trauma exposure in childhood. Future work should aim to optimize the implementation of high-quality mindfulness programs in youth populations. Further research should explore the mechanisms of mindfulness and the long-term outcomes of mindfulness interventions in childhood into adulthood, as well as outcomes in offspring. 

## Figures and Tables

**Table 1:** Beneficial outcomes seen in research of mindfulness programs for children and youth.

Outcome	Reference
Decreased anxiety	Sibinga et al., 2013 [78]
	Jee et al., 2015 [80]
Decreased rumination	Sibinga et al., 2013 [78]
Decreased school related stress, coping with stress	Sibinga et al., 2013 [78]
	Jee et al., 2015 [80]
Flatter cortisol curve	Sibinga et al., 2013 [78]
Lower levels of somatization	Sibinga et al., 2016 [79]
	Biegel et al., 2009 [87]
Decreased depressive symptoms	Sibinga et al., 2016 [79]
	Kuyken et al., 2013 [83]
Effectiveness in social gains	Jee et al., 2015 [80]
Classroom behavior	Black et al., 2015 [86]
	van de Weijer-Bergsma et al., 2012 [85]
Decreased hostility	Biegel et al., 2009 [87]
	Sibinga et al., 2016 [79]
Decreased suicidal ideation	Britton et al., 2014 [84]
Decreased self-harm	Britton et al., 2014 [84]
Reduced child abuse potential by parents	Dawe and Harnett, 2007 [95]
Conflict avoidance	Sibinga et al., 2014 [81]
Improved attention	van de Weijer-Bergsma et al., 2012 [85]
Greater well-being	Kuyken et al., 2013 [83]
Decreased post traumatic symptoms severity	Sibinga et al., 2016 [79]

**Table 2:** Variations in mindfulness programs and implementation.

Population	Program	Instruction	Setting	Duration	Reference
Public urban middle school (5th–8th grade; avg. 12 years), *n* = 300	Adapted MBSR	Trained MBSR instructors and personal practice 10+ years	School	12 weekly 50-min sessions	Sibinga et al., 2016 [79]
Foster care youth ages 14–21, *n* = 42	Adapted MBSR	Psychologist with expertise in mindfulness, two pediatrician lead group activities	Conference room of a joint family visitation and clinic space	10 weekly 2-h sessions	Jee et al., 2015 [80]
Kindergarten through 6th grade, low-income and ethnic minority students, *n* = 409	Mindful Schools (MS) Program or MS Plus	MS instructor with 3–20 years mindful meditation experience and classroom teacher facilitated	School	5-week (MS) or 7-week (MS Plus), 15-min sessions running three times per week	Black and Fernando, 2014 [118]
Ages 13–21, underserved youth, *n* = 43	Adapted MBSR	Instructors trained in MBSR, 10+ years’ experience	Primary care pediatric clinic	8 weekly 2-h sessions	Sibinga et al., 2014 [81]
Middle-school, urban youth, *n* = 41	Adapted MBSR	Trained MBSR instructor, 10+ years’ experience	School	12 weekly 50-min sessions	Sibinga et al., 2013 [78]
Adolescents ages 12–16 years, multiple schools, *n* = 522	School teacher facilitated adapted MBSR (Mindfulness in Schools Project – UK)	Teachers trained by instructors with MBSR training	School	9-week	Kuyken et al., 2013 [83]
6th grade students, *n* = 101	Meditation instruction and student writing exercises	One teacher with meditation training and 5+ years’ experience, one teacher who completed MBSR course, no experience	School	Daily for 6 weeks	Britton et al., 2014 [84]
Adolescents ages 14–18, *n* = 104	Adapted MBSR	Instructors MBSR trained	Outpatient psychiatric facility	8-week, 2 h/week, home 20–25 min homework daily	Biegel et al., 2009 [87]
Age 11–15 years, with ADHD, parents, and tutors; *n* = 38	Adapted MBSR combining Mindfulness in Schools Project, and methods for children with ADHD	Instructors trained in MBSR	Group program at an academic treatment center	8-week	van de Weijer-Bergsma et al., 2012 [85]
Ages 13–21, HIV infected, *n* = 11	Adapted MBSR	Instructor trained in MBSR, prior experience	Group at specialty HIV clinic	Eight 2-h sessions and a 3-h retreat	Sibinga et al., 2008 [82]

MBSR, Mindfulness Based Stress Reduction; ADHD, attention-deficit/hyperactivity disorder; HIV, human immunodeficiency virus.

**Table 3:** Examples of structured mindfulness programs for children.

Program Title	Website
Inner Resilience Program	http://www.innerresilience-tidescenter.org/
Wellness and Resilience Program	http://sbsd.schoolfusion.us/modules/cms/pages.phtml?pageid=195404&SID
Mindful Schools	www.mindfulschools.org
Learning to Breathe	www.learning2breathe.org
Mindfulness in Schools Project (“.b”, or “Stop and Be!” curriculum)	www.mindfulnessinschools.org
Still Quiet Place	www.stillquietplace.com
Stressed Teens	www.stressedteens.com
Wellness Works in Schools	www.wellnessworksinschools.com
Center for Mindful Awareness	www.centerformindfulawareness.org
